# Phosphatidic acid, phospholipase D and tumorigenesis^☆^

**DOI:** 10.1016/j.jbior.2013.08.006

**Published:** 2013-09-19

**Authors:** Julian Gomez-Cambronero

**Affiliations:** Department of Biochemistry and Molecular Biology, Boonshoft School of Medicine, Wright State University School Medicine, 3640 Colonel Glenn Highway, Dayton, OH 45435, USA

## Abstract

Phospholipase D (PLD) is a membrane protein with a double role: maintenance of the structural integrity of cellular or intracellular membranes and involvement in cell signaling through the product of the catalytic reaction, PA, and through protein–protein interaction with a variety of partners. Cross-talk during PLD signaling occurs with other cancer regulators (Ras, PDGF, TGF and kinases). Elevation of either PLD1 or PLD2 (the two mammalian isoforms of PLD) is able to transform fibroblasts and contribute to cancer progression. Elevated total PLD activity, as well as overexpression, is present in a wide variety of cancers such as gastric, colorectal, renal, stomach, esophagus, lung and breast. PLD provides survival signals and is involved in migration, adhesion and invasion of cancer cells, and all are increased during PLD upregulation or, conversely, they are decreased during PLD loss of function. Even-though the end results of PLD action as relates to downstream signaling mechanisms are still currently being elucidated, invasion, a pre-requisite for metastasis, is directly affected by PLD. This review will introduce the classical mammalian PLD’s, PLD1 and PLD2, followed by the mechanisms of intracellular regulation and a status of current investigation in the crucial involvement of PLD in cancer, mostly through its role in cell migration, invasion and metastasis, that has grown exponentially in the last few years.

## Introduction

**Fig. 1:**
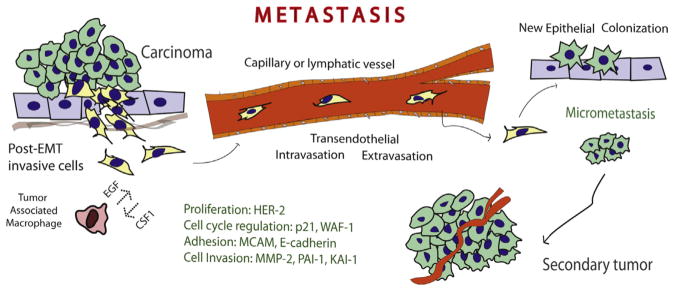
The process of metastasis. Post-EMT invasive cells leave the primary tumor and enter the circulatory system via trans-endothelial intravasation where the primary tumor cells migrates to a capillary or the lympatic system and then exits the circulation using transendothelial extravasation. At this point, the migrated epithelial cells colonize new tissue and become micrometastases that eventually develop into full blown tumors.

The role of Phospholipase D (PLD) in cancer and tumorigenesis has been studied in detail in the last decade. Elevated PLD activity, as well as overexpression was reported in a wide variety of cancers such as gastric, colorectal, renal, stomach, esophagus, lung and breast. Mitogenic and survival effects have been observed and are associated with an elevated expression of PLD. Among all these effects, one stands out, that is the role of PLD in cell migration. PLD involved in protein–protein interaction with a large variety of signaling molecules (Grb2, Rac2, WASP, S6K and JAK3, among others) or via phosphatidic acid (PA) is at the center of a complex signaling network that accelerates cell movement. PLD overexpression increases cancer cell invasion and metastasis. The process of metastasis is multifaceted and is presented schematically in Fig. 1. This review will introduce the classical mammalian PLD’s, PLD1 and PLD2, followed by the mechanisms of intracellular regulation and a status of the scientific literature in the crucial involvement of PLD in cancer, mostly through its role in cell migration, invasion and metastasis.

## What PLD does

PLD is a hydrolase that breaks the phospholipid, phosphatidylcholine (PC) to choline and PA. PLD is a membrane protein with a double role: maintenance of the structural integrity of cellular or intra-cellular membranes (Frohman et al., 1999) and involvement in cell signaling through the product of the catalytic reaction, PA, and through protein–protein interaction with a variety of partners (such as small GTPases, Kinases and phosphatases). The conversion of PC to PA by PLD in general is dependent on the presence of the co-factor phosphatidylinositol 4,5-bisphosphate (PIP_2_). PIP_2_ plays a role in both Arf-regulated PLD activity, as well as Rho-regulated PLD activity via potential docking of PLD to the plasma membrane (Hammond et al., 1997).

Development of potent isoform-specific small-molecule PLD inhibitors is integral to the advancement of the PLD field. Until recently, many PLD inhibitors lacked isoform specificity and did not act directly on the lipase. Halopemide and its subsequent derivative 5-fluoro-2-indoyl deschlorohalopemide (FIPI) have been found to be very effective inhibitors of PLD-mediated F-actin cytoskeleton reorganization, cell spreading and chemotaxis (Su et al., 2009). Use of iterative analog library synthesis approaches coupled with biochemicals assays and mass spectrometric lipid profiling of cellular responses has given rise to the next generation of halopemide derivatives, which have yielded the development of dual PLD1/2, PLD1 selective and PLD2 selective inhibitors (Lewis et al., 2009). Small molecules that directly inhibit PLD1 or PLD2 represent novel approaches for the investigation of potential treatment of metastatic cancer and inflammatory diseases (Lavieri et al., 2009, 2010; Lewis et al., 2009; Scott et al., 2009).

## The HKD phospholipase signature

**Fig. 2:**
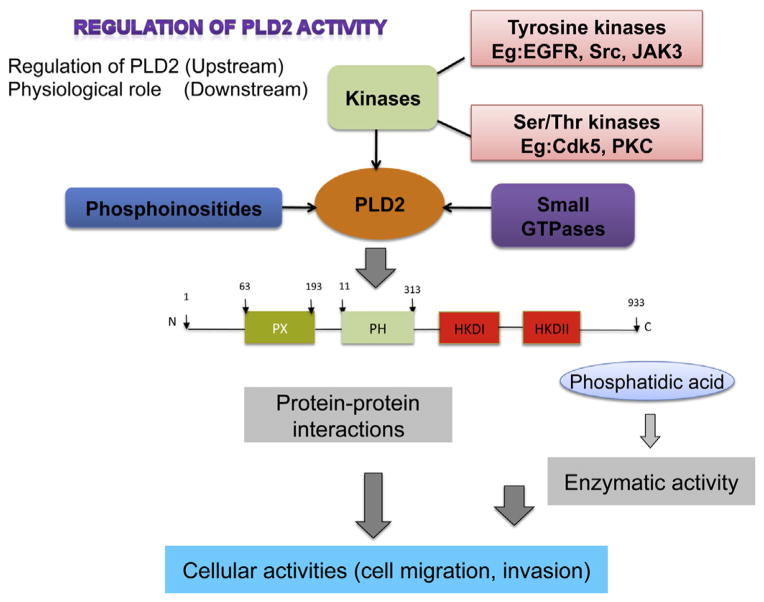
Regulation of PLD2. Tyrosine and/or serine threonine kinases act upstream of PLD and activate it by phosphorylation of certain targeted residues. Phosphoinositides and small GTPases also act on PLD2 signaling pathways. All of these upstream actions ultimately control PLD2’s ability to interact with other protein partners via protein–protein interactions and also modulate its lipase and GEF activities, which effect the downstream targets such as chemotaxis and cell invasion.

There are two mammalian isoforms of the gene that have been cloned from human and murine sources, *PLD1* and *PLD2*, which yield the PLD1 and PLD2 proteins and four slightly shorter splice variants (Fig. 2). PLD genes undergo qualitative and quantitative changes in transcriptional upregulation during granulocytic differentiation of HL-60 cells. The PLD1 gene has been localized to the long arm (q) of chromosome 3 (3q26) (Park et al., 1998a), covers 210 kb of genomic DNA that is defined by 31 exons, whereby 27 exons result in the expression of four splice variants (PLD1a, PLD1a2, PLD1b and PLD1b2) (Katayama et al., 1998; Hammond et al., 1997). The mammalian PLD2 gene is found on the short arm (p) of chromosome 17 (17p13) (Park et al., 1998b), is defined by 25 known exons of a genomic region spanning 16.3 kb and encodes for two splice variants (PLD2a and PLD2b) of 933 amino-acids in length each (Steed et al., 1998).

All members of the PLD superfamily possess two highly conserved phosphatidyltransferase HKD catalytic domains (HKD1 and HKD2) that are defined by the consensus peptide sequence HxK(x)4D(x) 6GSxN, which are vital to the lipase activity. The PX domain has been heavily implicated in binding to certain regulatory factors (PIP) and proteins (growth factor receptor-bound protein 2 (Grb2) and epidermal growth factor receptor (EGF-R), while the PH domains of PLD1 and PLD2 have been demonstrated to function as strong modulators of the membrane recycling machinery that results in regulated growth factor receptor endocytosis and also linked to binding to SH2/SH3-containing tyrosine kinases (Sorkin, 2001).

## Role of small GTPases in PLD signaling

PLD has been associated with a variety of physiological cellular functions, such as cancer cell progression, intracellular protein trafficking, cytoskeletal dynamics, membrane remodeling and cell proliferation in mammalian cells and meiotic division and sporulation in yeast. The PLD2 isoform is at the center of a complex network of signaling proteins (Fig. 2). PLD2 can be activated in intact cells by a variety of agonists and tyrosine kinases (Foster and Xu, 2003) and can be regulated by small GTPases and certain PKC family members (Du et al., 2000). PLD2 and Rac2 physically interact and hetero-dimerize *in vitro*, and recently, the biphasic effect of a monomeric GTPase acting as a master switch has been shown to both promote and inhibit phospholipase activity as relates to the timeline of chemotaxis (Peng et al., 2011). Macrophages that overexpressed both Rac2 and PLD2 experienced a strong initial response towards the chemoattractant that was significantly decreased at later time points. This initial positive response was attributed to the presence of a PLD2-Rac2 positive feedback loop, while the subsequent negative response of Rac2 on PLD2 was confirmed using cells from Rac2^−/−^ mice that exhibited increased PLD2 enzymatic activity, which was reversed by PIP_2_. It has been hypothesized that a Rac2-mediated inhibition of PLD2 function occurs because of Rac2 sterical interference with the PH domain membrane binding site of PLD2 and ensuing PIP_2_ deprivation (Peng et al., 2011). Rac2 localized *in vivo* to the leading edge of leukocyte pseudopodia with PLD2 being physically posterior to this wave of Rac2. Both PLD2 and PA signal to DOCK2, which mediates Rac activation and actin modeling (Nishikimi et al., 2009).

## Tyrosine kinases and phosphatases as key regulators of PLD

There are many reports indicating a regulation of PLD by tyrosine kinases and phosphatases. Choi et al. (2004) have found that PLD2 is specifically phosphorylated on residues Y11, Y14, Y165 and Y470. Mutation of Y470 resulted in a 50% decrease in PLD2 activation and suggests some partial loss of catalytic activity. Additionally, mutation of only Y14 and not the other three tyrosine residues yielded mislocalization of PLD2 when using immunofluorescence microscopy. Recently, phosphorylation targets within the PLD2 molecule have been mapped that are vital to its regulation as a lipase and thus correlated *in vitro* to at least 3 different tyrosine kinases: EGF-R, Src and Janus Kinase 3 (JAK3) (Gomez-Cambronero, 2010; Henkels et al., 2010). Using LC-MS analyses to prove the presence of phospho-PLD2-peptides, the specific PLD2 tyrosine residues phosphorylated by these kinases are Y296, Y511 and Y415, respectively, that yield either positive or negative effects on the lipase. PLD2 but not PLD1 physically complexes with and interacts with the intracellular part of the EGF-R in a ligand-independent manner following receptor activation. Elevation of either PLD1 or PLD2 has the potential to transform rat fibroblasts and contribute to cancer progression of the malignant phenotype in cells that also have elevated levels of EGF-R or Src tyrosine kinases (Foster and Xu, 2003).

The potential exists for stimulation of PLD activity to directly contribute to cell proliferation, which further compounds the formation of a fully malignant phenotype (Foster and Xu, 2003). Contrarily, it has been hypothesized that PLD2 activity in certain breast cancer cell lines is comparatively low compared to non-cancerous cells or other breast cancer cell lines because it is downregulated by tyrosyl phosphorylation at Y296 via EGF-R (Gomez-Cambronero, 2010), which can also be correlated to a negative impact on the relative levels of cell invasiveness of these breast cancer cells (Foster and Xu, 2003). This low level of PLD activity can be increased by *in vitro* treatment with either JAK3 or Src (Gomez-Cambronero, 2010). Src participates in the activation of PLD through the Ras pathway and the kinases Fyn and Fgr but not Lyn (Choi et al., 2004).

## Role of phospholipase D in cancer

PA, the product of PLD enzymatic action, is a major lipid second messenger that regulates a plethora of signaling pathways and cellular functions such as chemotaxis and cell proliferation. Apart from being a regulator of these physiologically essential processes, PLD plays a role in tumorigenesis. Elevated PLD activity as well as expression was reported in wide variety of cancers such as gastric, colorectal, renal, stomach, esophagus, lung and breast.

Increased PLD2 expression and activity is observed in human colorectal cancer when compared to normal mucosa (Oshimoto et al., 2003). In addition, a PLD2 gene polymorphism was shown to be prevalent in colorectal cancer in a Japanese case study, where it was demonstrated that a C → T mutation resulting in Thr → Ile is associated with colorectal cancer. However, lipase activity was not effected with this mutation (Yamada et al., 2003). In yet another study, 97 colorectal carcinomas that were obtained from surgeries were examined. An increase in PLD2 levels was reported, in which PLD2 expression varied from tumor to tumor. An obvious correlation was observed between PLD2 expression and the tumor size as well as patient survival. This study suggested PLD2 might be a prognostic indicator in colon cancers (Saito et al., 2007). PLD1 was also shown to be involved in tumor progression. Tissue microarrays with 122 human colon cancer tissues showed positive staining for PLD when compared to normal mucosa. In the same study, it was found that PMA induces PLD1 rather than PLD2 in a Raf/ERK- and NFkb-dependent fashion, which further induces MMP-9 production (Kang et al., 2008).

Melanoma cell lines, but not primary melanocytes, have high levels of PLD activity that is dependent on PKC, Rho and phorbol ester, suggesting that it is PLD1 rather than PLD2 that is involved in cancer progression (Riebeling et al., 2003). Protein kinase Czeta activation by PLD2-PX domain promotes survival of breast cancer cells (Kim et al., 2005). PLD’s role was also observed in breast cancer cell lines. Highly aggressive, estrogen receptor negative (ER-ve) MDA-MB-231 cells possess higher levels of PLD1 protein, and therefore, these cells have 8-fold higher lipase activity when compared to low invasive MCF-7 cells, which have relatively low levels of PLD1. Cell viability experiments revealed that PLD prevents apoptosis and acts as survival signal for the ER-ve cells (Zhong et al., 2003). It has recently been reported that PLD2 positively contributes to the invasive phenotype of v-Src-transformed cells (Shen et al., 2002). Table 1 compiles some work on the implication of the PLD2 isoform in cancer.

### PLD provides strong survival signals

There are multiple mechanisms by which PLD-mediated survival signals are generated in cancer cells, which are discussed below. PLD suppresses phosphoprotein 2A (PP2A), reduces its association with E4BP and S6K and aids in transformation of cells (Hui et al., 2005). PLD activity correlates with rapamycin resistance of breast cancer cells. The human breast cancer cell line MDA-MB-231 with high levels of PLD are more resistant to rapamycin when compared to MCF-7 cells that possess low PLD levels. In a subset of breast cancer cells that are negative for phospho Akt, the PLD1/mTOR pathway is active, suggesting that PLD1 is a major survival signal in this scheme. Hence, it was suggested that PLD1 could be used as a biomarker for rapamycin based therapies (Chen et al., 2005; Gozgit et al., 2007). PLD2 interacts with mTOR and activates it, which provide survival signals (Ha et al., 2006). In MCF-7 cells, PLD as well as the estrogen receptor (ER) promote survival by preventing proteasomal degradation of myc (Chen et al., 2003). PLD in MDA-MB-231 cells stabilizies mutant p53 in these cells in a MAPK-dependent manner. In turn, PLD-generated survival signals dependent on mutant p53 (Hui et al., 2006).

PLD also acts as a survival signal for cancers, such as renal cancer cells where PLD regulates hypoxia inducible factor 1α (HIF-1 α) at the translation level, in a vHL-independent fashion and promotes cancer cell proliferation (Toschi et al, 2008). In ovarian cancer cells, PLD is shown to be essential for agonist-induced LPA production and promotes motility, growth and proliferation (Luquain et al., 2003). Another mechanism by which PLD promotes cancer growth is by preventing apoptosis of cancer cells. PLD2 promotes survival of stomach cancer cells by preventing apoptosis (Cho et al., 2008). PLD2 enhances the expression of anti-apoptotic proteins, such as Bcl-2 and Bcl-xL in lymphoma cells (Oh et al., 2007).

### PLD is involved in migration, adhesion and invasion of cancer cells

In addition to being a survival signal, elevated PLD also provides migration cues in several cancers, such as bladder, lung, skin and breast carcinoma (Zheng et al., 2006). Elevated PLD is associated with MMP9 release in an MAPK/NFKβ-dependent pathway in an acidic environment, which mimics the tumor microenvironment (Kato et al., 2005). While inhibition of PLD abrogated MMP9 secretion, addition of PA rescued norepinephrine-induced MMP9 secretion that is crucial for tumor metastasis and invasion (Taves et al., 2008). PLD2’s lipase activity is implicated in migration and invasion of lymphoma cells via focal adhesion kinase (FAK) activation (Knoepp et al., 2008).

### Cross-talk of PLD signaling with other cancer regulators (Ras, PDGF, TGF and kinases)

Fibroblasts lacking PLD1 activity could form tumors only in the presence of PA indicating that PLD1 is necessary for Ras-mediated transformation (Buchanan et al., 2005). Cancer cells with increased Ras activation in a RalA/PI3K-dependent manner enhances PLD activity, which in turn provides survival signals (Shi et al., 2007). PLD2 is linked to the progression of EWS-Fli sarcoma due to its cross-talk with PDGF-mediated signaling (Nozawa et al., 2005). A transmodulation between PLD2 and the oncogenic kinase RET is evident in thyroid cancer cells where PLD2 enhances STAT3 phosphorylation and transcriptional activation that is mediated by RET (Kim et al., 2008). In addition, PLD inhibition increases TGFβ signaling and TGFβ-mediated increase in cyclin kinase inhibitors, p21Cip and p27Kip, implicating PLD in suppression of TGFβ signaling (Gadir et al., 2007). The dephosphorylation mutant of PLD2, PLD2-Y179F, is known to enhance DNA synthesis by activating MEK and Akt, suggesting a role for kinase-mediated regulation of PLD2 in cell proliferation (Di Fulvio et al., 2008). Interestingly, PLD activity is also involved in virus-induced carcinoma. Hepatitis C virus core protein transforms NIH3T3 cells in a PLD-dependent fashion (Kim et al., 2004).

### Recent developments in cancer and PLD research

**Fig. 3:**
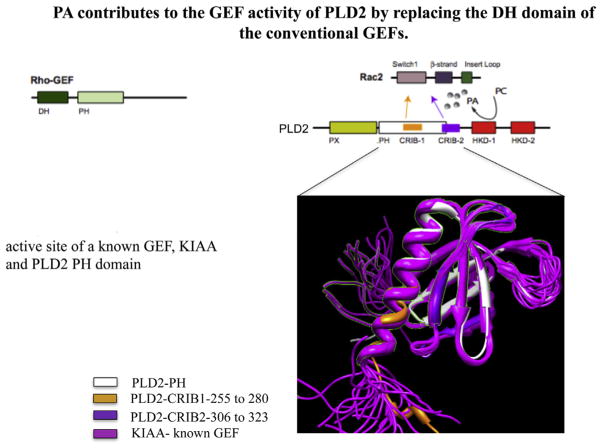
PLD2 as a GEF. We modeled PLD2 structural domains and found that its PH domain aligns with the PH domain of the Rac-GEF, KIAA/SWAP70, including portions where PLD2 can bind to Rac2 through its CRIB domains.

The last 5–6 years have witnessed an exponential growth in research in PLD and cancer. A gene called FAM83B which is involved in the transformation of cells increases the amount of PLD activity (Cipriano et al., 2013). When PLD1 was silenced, the growth of cells was slower than in the mock condition. It also was found that breast cancer cells (MCF-7 and MDA-MB-468) that are dependent on FAM83B expression to grow are sensitive to PLD inhibitors. FAM83B expression also increases EGFR activity. When PLD2 was silenced in MDA-MB-231 cells, which were transplanted into mice, primary tumor size was reduced when compared with normal tumors (Henkels et al., 2013b). The inverse was also found to be true when PLD2 was overexpressed in the MCF-7 cells. PLD2 has been reported to have a Guanine-nucleotide Exchange Factor (GEF) activity for Rac (Fig. 3) (Mahankali et al., 2011) and for Rho (Jeon et al., 2011) small GTPases, which is implicated in cell migration.

PLD inhibitors showed a negative effect on tumor growth in mice as well. There are six single nucleotide polymorphisms (SNP) of PLD1 in non-small cell lung cancer (Ahn et al., 2012). These six SNPs were located in the catalytic domain of PLD1. PLD inhibitors that were found to decrease the invasion of glioblastoma cells were found by the HA Brown lab (O’Reilly et al., 2013). A PLD2-specific inhibitor (ML298) and a dual PLD1/PLD2 inhibitor (ML299) were both found to have a potential role in treating brain cancer. Fes and JAK3 were found to elevate PLD2 expression and this interaction was found to be a reason for the elevated proliferation rate of MDA-MB-231 cells (Ye et al., 2013). Also involving JAK3, starvation of cells showed an increase in PLD2 activity and apigenin was shown to inhibit the positive effect of JAK3 in starved cells (Ye et al., 2012). Additionally, cell invasion of MTLn3 cells is dependent on PLD2 and JAK3 (Henkels et al., 2011).

The lack of PLD1 decreased the number of metastases and tumor size (Chen et al., 2012). Also, the lack of PLD1 decreased the vascularization of the tumors. It was found that PLD2 binds to Ras and acts as a GEF, which can cause increased cell growth (Henkels et al., 2013b). Ras signaling was due to increased PLD1 activity which was the result of phosphoprotein enriched in astrocytes 15 kDa (PEA-15) expression (Sulzmaier et al., 2012). PEA-15 promotes G1- to S-phase transition in cells. Inhibiting PLD1 or interfering with the binding of PLD1 to PEA-15 reduced the activation of Ras. Quercetin, a flavonoid that is known to downregulate matrix metalloproteinase protein 2 and 9 (MMP-2 and MMP-9), which are found at increased levels in tumors, downregulates PLD1 activity in these cancerous cells (Park and Min Do, 2011). Overexpression of PLD contributes to tumor progression through MMP-2 transcription.

Silencing PLD blocked the ability of β-catenin to activate PLD and other Wnt genes to form the β-catenin/TCF-4 complex (Kang and Min do, 2010). Elevating the levels of PA in the cells was found to enhance the formation of that group, which is necessary for colorectal tumors and are driven by the Wnt/β-catenin pathway and a negative effect on PLD1 and PLD2 expression in AGS and MKN-1 gastric cancer cells (Kang et al., 2010). Not only did the PLD knockdown reduce the amount of inflammation, it also reduced the amount of proliferation of gastric cancer cells. Breast cancer cells (SK-BR3) had increased invasion due to a similar PLD1/MMP model (Kang et al., 2011). A mutation of Ras resulted in increased levels of PLD1 mRNA in colon cancer cells (Gao et al., 2009) and the mutated-Ras interacts with PLD1 via the Sp1 transcription factor.

A potential therapeutic target for osteolytic bone metastases in lung cancer patients has been proposed (Hsu et al., 2010). The authors have identified PLD’s involvement in IL-8 mediated PLD/PKC/ERK1/2 signaling. Inhibiting PLD resulted in lower amounts of osteoclast formation by inhibiting protein kinase C’s activation and thereby stopping the phosphorylation of ERK1/2 (extracellular signal-regulated kinase 1/2). PLD inhibitors clearly inhibit the invasion of breast cancer cells in culture (Su et al., 2009) and, similarly, inhibition of PLD1 and PLD2 by triptolide decreases cell proliferation in MDA-MD-231 cells (Kang et al., 2009). A review by David Foster indicates that elevated PLD expression can cause rapamycin resistance in the mammalian target of rapamycin (mTOR) (Foster, 2009). Since PLD expression is elevated in most cancer cells the production of PA is increased. PA is a competitor with rapamycin and when PLD activity is elevated, rapamycin resistance can be conferred. Since rapamycin is commonly used in therapies, PA and PLD activity needs to be considered.

PLD couples survival and migration in tumor cell lines (Zheng et al., 2006). Overexpression of wild-type PLD2 has been implicated in EL4 lymphoma metastasis *in vivo*, while overexpression of catalytically inactive PLD2 generated fewer liver metastases compared to control cells (Knoepp et al., 2008). Recently, Chen et al. examined the *in vivo* role of PLD1 in melanoma growth and metastasis, showing that administration of the inhibitor FIPI into wild-type mice or the loss of PLD1 via PLD1 knockout mice led to a significant reduction of tumor metastases. These results implicate the importance of PLD1 in the tumor microenvironment, which aids in tumor growth/metastasis (Chen et al., 2012).

In our lab, we have recently demonstrated that PLD2 plays a role in breast cancer invasion and tumorigenesis *in vivo* (Henkels et al., 2013b). PLD2 was stably silenced in highly invasive breast cancer cells and led to tumors derived from these cells being only mildly invasive in SCID mice. Conversely, when PLD2 was overexpressed in a low invasive breast cancer cell line and xenotransplated into SCID mice, more substantial breast tumors arose. Moreover, implanting Alzet pumps containing PLD-specific inhibitors into SCID mice led to a reduction in the number of breast tumors and overall metastasis following xenotransplantation. We determined the mechanism of mammary tumor cell invasion and metastasis seen following PLD2 overexpression as being mediated by PA, Grb2 and Rac2. PLD2 is a key factor for cell invasion that contributes critically to growth and metastasis of breast tumors *in vivo*, which have clear pharmacological implications in humans.

## Conclusions

PLD regulation in cells occurs via two different signaling pathways. One is via growth factors/mitogens, such as EGF, PDGF, insulin and serum and implicates tyrosine kinases. This pathway involves interactions with Grb2, Sos and the kinases EGF-R, JAK3 and Src. The other pathway is via the small GTPases, such as Arf, Rac2 and Rho, and is directly related to cell migration, a process in which PLD plays a vital role. Eventhough the end results of PLD action as relates to downstream signaling mechanisms are still currently being elucidated, cell migration and cell invasion are modulated directly by PLD. Invasion is a requisite for metastasis, and as such, the interest in PLD in metastasis has spiked in the last few years. Mitogenic and survival effects are seen in angiogenesis, tumor development, growth and metastasis, and all are increased during PLD upregulation or decreased during PLD loss of function. Small molecules that either indirectly or directly inhibit PLD1 or PLD2 represent novel approaches for the investigation of potential treatment of metastatic cancer. The status of current investigation in the crucial involvement of PLD in cancer, mostly through its role in cell migration, invasion and metastasis, has grown exponentially in the last few years, and is expected to continue growing and yielding more exciting results.

## Figures and Tables

**Table 1:** Role of PLD2 in cancer signaling. Recent discoveries documenting the increasing importance of the PLD2 isoform as a crucial component of cancer signaling.

Phospholipase D2 (PLD2) and cancer
PLD2 contributes to the invasive phenotype of v-Src-transformed cells (Shen et al., 2002). Elevated PLD activity confers rapamycin resistance and survival signals in human bladder and lung cancer cells (Shi, 2007; Chen, 2003). Protein kinase Czeta activation by PLD2-PX domain promotes survival of breast cancer cells (Kim et al., 2005). The presence of elevated mutant p53 levels contributes to the cell survival signal that occurs as a result of high PLD activity (Hui et al., 2006). Increased expression of PLD2 correlates well with increased tumor size and increased mortality in humans (Saito M et al., 2007). Expression of PLD2 enhances processes favorable to lymphoma cell metastasis (Knoepp SM et al., 2008). Cell invasion of MTLn3 cells is dependent on PLD2 and JAK3 (Henkels et al., 2011). PLD2 overexpression leads to early breast cancer onset and larger lung metastasis. Silencing PLD2 does the opposite (Henkels et al., 2013).
